# Effect of frailty on medication deviation during the hospital-family transition period in older patients with cardiovascular disease: An observational study

**DOI:** 10.1097/MD.0000000000036893

**Published:** 2024-01-12

**Authors:** Meng-Yao Liang, Li Feng, Wuyang Zhu, Qing-Qing Yang

**Affiliations:** a Department of Nursing, The Sixth People’s Hospital of Nantong, Jiangsu, China; b Department of Rehabilitation, Yi Jiangmen Community Health Service Center, Gulou District, Nanjing, China; c Department of Cardiology, The Sixth People’s Hospital of Nantong, Jiangsu, China.

**Keywords:** an observational study, cardiovascular diseases, frailty

## Abstract

Studies have shown that frailty increases cardiovascular disease (CVD) incidence in older patients and is associated with poor patient prognosis. However, the relationship between medication deviation (MD) and frailty remains unclear. This study aimed to explore the influence of frailty on MD during the hospital-family transition period among older patients with CVD. Between February 2022 and February 2023, 231 older people CVD patients were selected from a class III hospital in Nantong City using a multi-stage sampling method. A general information questionnaire was used to collect the socio-demographic characteristics of the participants prior to discharge, the frailty assessment scale was used to assess the participants frailty, and a medication deviation instrument was used to assess the participants MD on the 10th day after discharge. Propensity score matching was used to examine the effect of frailty on MD in older patients with CVD during the hospital-family transition period. The incidences of frailty and MD were 32.9% (76/231) and 75.8% (175/231), respectively. After propensity score matching, the risk of MD in frail patients with CVD was 4.978 times higher than that in non-frail patients with CVD (95% CI: [1.616, 15.340]; *P* = .005). Incidences of frailty and MD during the hospital-family transition period are high in older patients with CVD, and frailty has an impact on MD. Medical staff in the ward should comprehensively examine older patients with CVD for frailty and actively promote quality medication management during the hospital-family transition period to reduce MD occurrence and delay disease progression.

## 1. Introduction

East Asian countries are confronted with significant challenges of population aging, primarily due to low birth rates and longer life expectancies. According to projections for 2030, the proportion of individuals aged 65 years or older will increase to 18.1%. The region will encounter significant challenges due to the heightened vulnerability of the older people population to cardiovascular diseases (CVD).^[1]^ According to the estimates from the 2021 China Cardiovascular Disease Report, there are approximately 330 million Chinese patients suffering from cardiovascular diseases, which accounted for over 40% of deaths in China in 2019.^[2]^ Despite the significant potential of drug intervention in reducing the incidence and mortality rates of CVD, a systematic review study revealed that only 45% of hypertensive patients in Asia were adhering to their medication regimen, leading to premature death as a primary consequence.^[3]^Therefore, promoting proper medication adherence among patients has become an imperative issue within current medical care and healthcare systems aimed at delaying disease progression and alleviating global burden.^[4]^

Medication deviation, also known as medication differences (MD), refers to the difference between the actual medication consumed by the patient and the doctor’s prescription in cases of changes in the treatment place, such as admission, transfer, and discharge from the hospital, and has an incidence of 3.4 to 98.0%.^[5–7]^ MD not only increases the readmission rate of patients, resulting in poor prognosis, but is also the main cause of adverse drug events.^[8,9]^ The hospital-home transition period is a crucial period of transitional care for patients and includes up to 2 months before discharge to home.^[10,11]^ Ideally, the transition period should include a comprehensive transitional care plan; however, real-life evidence suggests that during the transition period, there is often a lack of continuity of related medication caused by reasons such as delayed information communication.^[12,13]^

The state of frailty is a multifactorial clinical condition characterized by reduced physiological reserve and heightened susceptibility to stressors, resulting in an increased risk of various adverse outcomes such as disability, hospitalization, and mortality. In the Chinese population aged over 65 years, the prevalence of frailty is approximately 10%.^[14]^ The prevalence of frailty among CVD patients in East Asia ranges from 25% to 60%,Frailty significantly increases the incidence of CVD and is strongly associated with a poor prognosis in older people individuals with CVD^[15,16]^ Nevertheless, the relationship between MD and frailty remains unclear. Whether frailty is involved in MD among older patients with CVD and the extent of its influence need further clarification. Therefore, this study examined the effect of frailty on MD during the hospital-family transition period of older patients with CVD in the Department of Cardiology of a tertiary hospital in Nantong.

## 2. Objects and methods

This was a cross-sectional study that was conducted in accordance with the ST rengthening the Reporting of OB servational studies in Epidemiology guidelines.^[17]^

### 2.1. Participants

Between February 2022 and February 2023, older patients diagnosed with CVD in the Cardiology Department of a class III geriatric hospital in Nantong were selected using a multi-stage sampling method. The diseases included were CVD due to atherosclerosis, that is, ischemic heart disease or coronary artery disease, and other CVDs, that is, hypertension, congenital heart disease, rheumatic heart disease, cardiomyopathy, and arrhythmia.^[18]^ The specific inclusion criteria were: Age ≥ 60 years; Self-care ability; and Informed consent to participate in this study. The exclusion criteria were: Day surgery patients;  < 2 Types of drugs prescribed at discharge; Transferred patients; Patients discharged voluntarily; Patients who died during hospitalization; Patients with mental disorders or inability to communicate; Patients who died during the follow-up period after discharge; and Patients who were followed up for 2 consecutive days and those who did not respond to telephone after 3 attempts. According to the sample size calculation method for observational studies, the sample size should be 5 to 10 times the number of independent variables.^[19]^ Sixteen independent variables were included in this study; hence, the sample size was estimated at 160 cases. Considering invalid questionnaires, the sample size was expanded by 10%^[20]^; therefore, a total of 231 samples were included in this study. This study was approved by the Ethics Committee of the Sixth People’s Hospital of Nantong (NO: NTLYLL 2022041).

### 2.2. Research methods

#### 2.2.1. General data collection.

A general information questionnaire was used to collect data on the participants social demographic characteristics, lifestyle indicators, and physical function status. The socio-demographic characteristics included sex, age, education level, marital status, living status, and economic status. The lifestyle indicators included smoking and alcohol consumption. Lastly, the physical function status included body mass index. The Charlson comorbidity index was used to evaluate the physical condition and visual function was evaluated by the evaluation standard of Chinese older people care service.^[21,22]^

### 2.3. Frailty status assessment

Using the FRAIL questionnaire that employs a frailty screening scale, frailty was assessed in the participants. This scale comprises 5 core domains and is a brief, interval-based screening tool, which includes slow movement, fatigue, low physical activity, comorbidities, and decreased physical fitness. Each item requires a “yes” or “no” response and is assigned a score of 1 or 0, respectively. The total score ranged from 0 to 5, with 0, 1 to 2, and 3 to 5 indicating no frailty, pre-frailty, and frailty, respectively.^[23]^

#### 2.3.1. Family family care index (APGAR) index.

APGAR is a subjective, quantitative evaluation tool for assessing the satisfaction of the family members with the family. The scale has 5 items, “usually” (2 points), “sometimes” (1 point), and “almost never” (0 points). The total score ranged from 0 to 10, with 7 to 10 indicating well-functioning families and 0 to 6 indicating moderately or severely dysfunctional families. The Cronbach α coefficient of the scale ranged from 0.80 to 0.88, and it has been widely used in Chinese families with good reliability and validity.^[24,25]^

#### 2.3.2. MD assessment during the hospital-family transition period.

The medication discrepancy tool (MDT) was developed by Smith^[26]^ to describe the MD during the nursing transition process and has been widely used during clinical nursing practice, including a Chinese study conducted in 2015. The Cronbach α coefficient for this tool was 0.97.^[27]^

#### 2.3.3. Self-efficacy assessment for rational medication intake.

The self-efficacy for appropriate medication use scale was developed by Risser et al^[28]^to measure patients medication self-efficacy. The higher the score on the scale, the higher the self-efficacy of medicine intake. If the total score rate is < 60%, the self-efficacy of medicine intake is poor. Dong Xiaofang translated the scale into Chinese, and the Cronbach α coefficient for the scale was 0.934.^[29]^

#### 2.3.4. Data collection and quality control.

Prior to the survey, 6 investigators received unified training, including mastering the use of CVD-related drugs, unification of the language expression of the terms used in the survey, and standardization of the communication methods. After the training, the consistency of the survey results of the investigators was checked, and a formal investigation was carried out after meeting the standard;

Data collection was divided into 2 parts. In the first part, the researchers obtained general baseline data of the patients by conducting a face-to-face questionnaire distribution on admission and evaluating the patients on the day of discharge using a questionnaire survey of APGAR, length of hospital stay, number of discharge drugs prescribed, and frailty scale score. The second part included a telephone follow-up using MDT tools on the 10th day after discharge (2 days in advance of the appointment by text message) to check whether the patient’s medication was consistent with the discharge doctor’s advice list, and data on deviation from the prescribed drugs were recorded. Caregivers were required to participate if patients were unable to follow-up.

### 2.4. Statistical methods

SPSS25.0 software(Armonk, NY: IBM Corp) was used for data statistics and analysis. To reduce the potential bias of covariates, we used the propensity score matching (PSM) method to include variables related to frailty, age, sex, smoking history, drinking history, diabetes, Charlson comorbidity index (CCI), and propensity score values were calculated by logistic regression analysis using a 1:1 nearest neighbor matching method without replacement, and the caliper value was set at 0.08. Continuous variables were described as mean ± standard deviation (χ̄±s), and categorical variables were described as relative frequencies and percentages. A *t* test was used for parametric tests, and the Mann–Whitney *U* test was used for nonparametric tests. Univariate and multivariate logistic regression analyses were performed to determine the influencing factors of MDT. A *P* value of < .05 was considered statistically significant.

## 3. Results

### 3.1. Frailty and MD in older patients with CVD during the hospital-family transition period

In this study, 255 patients were investigated. Among these, 9 patients who did not respond to the telephone follow-up after discharge, 6 patients who refused to be followed up, and 9 patients who had entered incomplete/inaccurate answers were excluded from the analysis. Finally, 231 patients (128 males and 103 females) were included, with a loss rate of 9.4%. Among these, 76 cases of frailty were identified, accounting for 32.9% of the included patients. Before matching, the MD incidence in older patients with CVD during the hospital-family transition period was 75.75% (175/231) with an average deviation of 2.48 ± 0.34 per person. The MD incidences in frail and non-frail patients were 86.84% (66/76) and 13.16% (10/76), respectively.

After 1:1 PSM, 40 pairs of patients were successfully matched. There were statistically significant differences in frailty, medication type, hospitalization days, family monthly income, diabetes, and CCI between the 2 groups before PSM (*P* < .05), and there were statistically significant differences in frailty, self-efficacy for appropriate medication use scale, and CCI after PSM (*P* < .05) (Table 1). Among them, the top 3 drugs with the incidence of single-type MD were pravastatin sodium tablets (50.94%, 54/106), aspirin enteric-coated tablets (46.2%, 67/165), and spironolactone (40.74%, 22/54) (Table 2). The top 3 types of MDs were missed drugs (25.78%, 148/574), timing errors (17.07%, 98/574), and drug substitution (9.58%, 55/574) (Table 3).

**Table 1:** General clinical characteristics of patients before and after PSM.

The project	Before PSM			After PSM		
	Non-md group (n = 56)	MD group (n = 175)	*P* value	Non-md group (n = 40)	MD group (n = 40)	*P* value
Age (yr)	74.30 ± 5.58	75.13 ± 5.18	.308	75.54 ± 5.52	76.13 ± 4.05	.581
Sex (%)			.543			.653
Female	23 (41.1)	80 (45.7)		17 (40.0)	19 (47.5)	
Male	33 (58.9)	95 (54.3)		23 (60.0)	21 (52.5)	
Marriage (%)			.735			.735
Married	49 (87.5)	156 (89.1)		36 (90.0)	34 (85.0)	
Divorced/widowed	7 (12.5)	19 (10.9)		4 (10.0)	6 (15.0)	
Education level (%)			.22			.443
Primary school and below	21 (37.5)	66 (37.7)		14 (35.0)	15 (37.5)	
Middle school and high school	15 (26.8)	75 (42.9)		10 (25.0)	14 (35.0)	
College or above	20 (35.7)	34 (19.4)		16 (40.0)	11 (27.5)	
Living situation (%)			.971			.955
Living alone	11 (19.6)	32 (18.3)		6 (15.0)	7 (17.5)	
Liv with spouse or children	42 (75.0)	134 (76.6)		32 (80.0)	31 (77.5)	
Living with other caregivers	3 (5.4)	9 (5.1)		2 (5.0)	2 (5.0)	
Smoking status (%)			.079			.499
Do not smoke	20 (35.7)	80 (45.7)		16 (40.0)	19 (47.5)	
Smoking	36 (64.3)	95 (54.3)		24 (60.0)	21 (52.5)	
Alcohol consumption (%)			.543			.654
Not drinking alcohol	33 (58.9)	95 (54.3)		20 (50.0)	18 (45.0)	
Drinking alcohol	23 (41.1)	80 (45.7)		20 (50.0)	22 (55.0)	
Status of frailty			.006			.002
Not frailty	46 (82.1)	109 (62.3)		31 (77.5)	17 (42.5)	
Frailty	10 (17.9)	66 (37.7)		9 (22.5)	23 (57.5)	
APGAR (%)			.446			.639
APGAR (0–6 points)	23 (41.1)	62 (35.4)		13 (32.5)	15 (37.5)	
APGAR(7–10 points)	33 (58.9)	113 (64.6)		27 (67.5)	25 (62.5)	
Type of medication (%)			.043			.654
≤5 types	29 (51.8)	64 (36.6)		20 (50.0)	18 (45.0)	
>5 types	27 (48.2)	111 (63.4)		20 (50.0)	22 (55.0)	
SEAMS (%)			.113			.040
Good	44 (78.6)	118 (67.4)		33 (82.5)	25 (62.5)	
Poor	12 (21.4)	57 (32.6)		7 (17.5)	15 (37.5)	
Days of hospitalization (%)			.007			.369
≤7 d	34 (60.7)	70 (40.0)		24 (60.0)	20 (50.0)	
>7 d	22 (39.3)	105 (60.0)		16 (40.0)	20 (50.0)	
Monthly household income per capita (%)			.029			.466
<1500 RMB	5 (8.9)	45 (25.7)		4 (10.0)	7 (17.5)	
1500–3000 RMB	14 (25.0)	35 (20.0)		9 (22.5)	11 (27.5)	
>3000 RMB	37 (66.1)	95 (54.3)		27 (67.5)	22 (55.0)	
Visual status(%)			.704			.887
No damage	39 (69.6)	123 (70.3)		28 (70.0)	26 (65.0)	
Mild impairment	15 (26.8)	49 (28.0)		11 (27.5)	13 (32.5)	
Moderate impairment	2 (3.60)	3 (1.70)		1 (2.5)	1 (2.5)	
Diabetes mellitus (%)			.016			.108
No	40 (71.4)	93 (53.1)		28 (70.0)	21 (52.5)	
Yes	16 (28.6)	82 (46.9)		12 (30.0)	19 (47.5)	
CCI	5.03 ± 0.76	5.42 ± 0.78	.002	5.25 ± 0.65	5.69 ± 0.51	.003

APGAR = family care index, CCI = Charlson comorbidity index, MD = medication deviation, PSM = propensity score matching, SEAMS = self-efficacy for appropriate medication use scale.

**Table 2:** Incidence of medication deviation for cardiovascular diseases.

Types of drugs	Drugs	Number of patients, n	Number of deviation, n	Incidence rate* (%)
Beta 1 blocking agent	Metoprolol sustained-release tablets	182	64	31.16
Reductase inhibitors	Pravastatin sodium tablets	106	54	50.94
	Rosuvastatin calcium tablets	57	19	33.33
Proton pump Inhibitors	Ilaprazole	97	20	20.62
Anticoagulants	Aspirin enteric-coated tablets	165	67	46.20
	Indobufen tablets	49	15	30.61
Calcium channel blockers	Amlodipine	122	29	23.77
Angiotensin II receptor antagonists	Irbesartan	144	33	22.91
Diuretics	Furosemide	101	35	34.65
	Spironolactone	54	22	40.74

* Incidence of single medication deviation (%) = (total number of single medication deviation)/(total number of all medication deviation) %.

**Table 3:** Incidence of medication deviation in elderly patients with CVD (n = 574).

Name of drug	Categories of medication deviation												
	A	B	C	D	E	F	G	H	I	J	K	L	Sum up, n
Metoprolol sustained-release tablets	26	4	0	8	21	11	1	0	6	0	9	0	86
Pravastatin sodium tablets	14	6	35	0	2	5	2	0	0	0	4	0	68
Rosuvastatin calcium tablets	4	3	13	1	2	0	0	0	1	0	1	0	25
Ilaprazole	5	2	0	3	1	0	3	0	4	0	5	2	25
Aspirin enteric-coated tablets	13	5	28	6	7	0	1	0	5	0	2	5	72
Indolebufen tablets	2	4	2	5	2	3	2	2	0	0	0	0	22
Amlodipine	17	1	4	5	2	2	4	1	0	0	0	2	38
Irbesartan	18	1	1	2	2	3	5	0	2	0	1	2	37
Furosemide	15	3	2	5	3	1	7	3	2	0	2	2	45
Spironolactone	13	5	1	2	4	2	4	2	0	0	1	3	37
Other	21	13	12	9	9	5	6	12	5	0	16	9	119
Sum up	148	47	98	46	55	32	35	20	25	0	41	25	574
Incidence rate %	25.78	8.19	17.07	8.01	9.58	5.57	6.10	3.48	4.36	0	7.14	4.35	100

A: missed drugs; B: irregular medication; C: time error; D: repeated medication; E: drug substitution; F: dose reduction; G: dose increase; H: decreased frequency; I: increased frequency; J: method error; K: self-withdrawal; L: increase in the variety.

CVD = cardiovascular disease.

### 3.2. Effect of frailty before PSM on MD during the hospital-family transition period in older patients with CVD

Variables with statistical significance among the general clinical data before PSM were included in the influencing factor analysis. The results of the univariate analysis of the influencing factors showed that frailty was associated with MD in older patients with CVD during the hospital-family transition period (OR = 2.785, 95% CI: 1.317, 5.891, *P* = .007). Furthermore, multivariate analysis showed that CCI was an independent risk factor for MD (OR = 1.784, 95% CI: 1.135–2.804, *P* = .012), and a monthly household income of > 3000 RMB was a protective factor for MD (OR = 0.342, 95% CI: 0.121–0.967, *P* = .043) (Table 4).

**Table 4:** Identification of MD influencing factors by univariate and multivariate logistic regression analyses before PSM.

Variables	Univariate logistic regression analysis		Multivariate logistic regression analysis	
	OR (95% Cl)	*P* value	OR (95% Cl)	*P* value
Frailty	2.785 (1.317, 5.891)	.006		
Type of medication > 5 kinds	1.863 (1.014, 3.421)	.043		
Days of hospitalization > 7 d	2.318 (1.252, 4.291)	.007		
Diabetes mellitus	2.204 (1.149, 4.228)	.016		
CCI	1.954 (1.278, 2.987)	.002	1.784 (1.135, 2.804)	.012
Monthly household income per capita 1500–3000 RMB	0.337 (0.116, 0.985)	.047		
Monthly household income per capita > 3000 RMB	0.296 (0.108, 0.813)	.018	0.342 (0.121, 0.967)	.043

Dependent variable: non-MD was set to 0, MD was set to 1; Type of medication ≤ 5 was used as the reference to set dummy variable, Hospitalization days ≤ 7 days was used as the reference to set dummy variables. Household per capita monthly income < 1500 RMB was used as the reference to set a dummy variable.

CCI = Charlson comorbidity index, MD = medication deviation, PSM = propensity score matching.

### 3.3. Effect of frailty after PSM on MD in older patients with CVD during the hospital-family transition period

The variables with statistical significance among the general clinical data after PSM were included in the influencing factor analysis. The results of the univariate analysis of the influencing factors demonstrated that frailty was associated with MD in older patients with CVD during the hospital-family transition period (OR = 5.221, 95% CI: 1.870, 14.520, *P* = .002). The results of the multivariate analysis further showed that frailty was a risk factor for MD in older patients with CVD during the hospital-family transition (OR = 4.978, 95% CI: 1.66–15.340, *P* = .005). Additionally, CCI was also a risk factor for MD in older patients with CVD during the hospital-family transition (OR = 4.652, 95% CI: 1.624–13.324, *P* = .004) (Table 5).

**Table 5:** Identification of MD influencing factors by univariate and multivariate logistic regression analyses after PSM.

Variables	Univariate logistic regression analysis		Multivariate logistic regression analysis	
	OR (95% Cl)	*P* value	OR (95% Cl)	*P* value
Frailty	5.211 (1.870, 14.520)	.002	4.978 (1.616, 15.340)	.005
CCI	3.872 (1.609, 9.314)	.003	4.652 (1.624, 13.324)	.004
SEAMS (poor)	3.116 (1.002, 9.498)	.040		

Dependent variable: non-MD was set to 0, MD was set to 1; SEAMS (good) was used as the reference to set the dummy variable.

CCI = Charlson comorbidity index, MD = medication deviation, SEAMS = Self-efficacy for appropriate medication use scale, PSM = propensity score matching.

## 4. Discussion

The objective of this study was to examine the impact and extent of frailty on medication deviation during the transition from hospital to home in older patients with CVD. Our findings revealed that both frailty and a higher comorbidity index were associated with an increased incidence of MD.

The incidence of MD is high in older patients with CVD during the hospital-family transition period. Medication safety is a major concern of the global healthcare system.^[30]^ In the present study, the MD incidence in the hospital-family transition period of older patients with CVD before the comparison was 75.75%, which was higher than the 61.89% incidence of MD reported by Zhao Linbo and lower than the 79.5% incidence of MD reported by Xue Wenjun.^[7,31]^ The variation in the incidence of MD may be related to the types of the disease and differences in the follow-up time of the MD.

In East Asia, the research on drug treatment in patients with CVD focuses on long-term adherence, medicine for the deviation the research problem is less.^[32,33]^ Some studies in developing countries have shown that patients taking cardiovascular medications during the transition period are more likely to suffer from medication bias, but the MD in CVD patients has not been specifically explored.^[34,35]^The variation in the incidence of MD may be related to the types of the disease and differences in the follow-up time of the MD. For example, pravastatin, the drug with the highest incidence of single-type MD (50.94%), needs to be taken before bed; hence, many patients easily forget to take the medicine and develop MD. Contrarily, problems such as inadequate medication and nursing, undetailed discharge education, and insufficient family support also contribute to MD. Despite the nurses providing written prescriptions and medication education at discharge, with time, patients gradually forgot the content of the drug education, thereby causing MD. In the later stages of clinical nursing, we should focus on the use of such drugs, communicate the prescription, and educate the patients thoroughly; continued management of drugs in the later stage of hospitalization improves the safety of the clinical medication.

Frailty is a risk factor for MD in older patients with CVD during the hospital-family transition period. The 2021 guidelines of the European Society of Cardiology emphasize the presence of frailty in patients with CVD; therefore, frailty has received special attention recently.^[36]^ The results of this study showed that frailty is a risk factor for MD in old people patients with CVD during the hospital-family transition period (OR = 4.978).This study weak incidence was 32.9%, in line with east Asia related research results.^[15,16]^ According to a study in Japan shown that frailty and CVD not only have collinearity but are also associated with an increased risk of cognitive deterioration in old people patients with CVD.^[37]^ This can also explain the effect of frailty on MD in old people patients with CVD. Contrarily, frailty can affect the patient’s cognitive status, creating problems in understanding drug dosage and intake. Furthermore, frailty combined with CVD increases the risk of polypharmacy, and some drugs, such as antiplatelet drugs, also promote frailty.^[38,39]^ Frailty screening should be included in routine health assessments for old people patients with CVD, especially those who are prescribed multiple drugs. Besides strengthening medication management, comprehensive intervention measures should be taken to improve their frailty status.

CCI is a risk factor for MD in old people patients with CVD during the hospital-family transition period. The results of the present study showed that CCI was a risk factor for MD in old people patients with CVD during the hospital-family transition period (OR = 4.652).With the aging of the population, the global burden of CVD disease will continue to increase, and most old people CVD patients have multiple comorbidities, and more than 70% of Asian patients with heart failure have more than 2 comorbidities.^[40,41]^In a large national survey of Medicare beneficiaries aged 65 years, diabetes (37–47%), anemia (39–51%), and arthritis (41–46%) were found to be commonly associated with CVD,^[42]^ and, consistent with those findings, the prevalence of diabetes was 42.42% in the present study. Management of multiple comorbidities often requires polypharmacy (usually defined as taking 5 or more medications).^[43]^ Moreover, the incidence of polypharmacy of ≥ 10 types of drugs was 69%, which was significantly higher than the incidence in the present study; this may be related to the racial differences in the population and the cultural differences at the medical level. Therefore, more attention should be paid to patients with multimorbidity in clinical nursing. On the 1 hand, these patients have multiple drug use problems, on the other hand, multiple comorbidities mean that they will be referred to different hospitals or different departments for treatment, which increases the probability of MD. Patients should be comprehensively evaluated to understand the CCI status in the early stage of discharge. Patients with higher CCI scores should be continuously tracked to improve their medication safety.

A systematic review demonstrated improvements in MD after the application of patient education, medication regimen management, prescription of fixed-dose combination medications, clinical consultation with pharmacists, and team care.^[44]^ Other strategies included cognitive behavioral therapy and the use of incentives and medication reminders, such as medication monitoring via text messages or reminders via email. However, these measures are usually used in foreign, high-income countries, and relevant domestic studies mainly focus on the barriers and influencing factors of MD; moreover, there are few relevant research strategies for reducing MD.^[7,31]^One of the main responsibilities of nurses in clinical work is safe drug management. Therefore, given the high incidence of MD, the importance of nurses medication safety ability is highlighted. In the later stage, not only should we strengthen the training of nurses’ pharmacy knowledge and improve their level of individualized education and medication communication for discharged patients but also actively explore the management of patients medication. Integrating with the “Internet +” platform to upload patients’ relevant medication information, which is conducive to patients self-check and for clinicians to make more reasonable medication decisions when there is a need for multi-hospital or multi-department treatment and improve patients’ medication safety more scientifically, should be considered.

## 5. Conclusion

In this study, the incidence of MD in old people patients with CVD within 10 days of discharge from the hospital-family transition period was 75.75%, and the incidence of frailty in clinical old people patients with CVD was 32.9%. The PSM study showed that frailty and Charlson comorbidity index were the main risk factors for MD. In clinical practice, we should strengthen the medication management of patients during the transition period, especially for patients with frailty who should receive key support from family members or caregivers within 10 days of discharge. Nurses should also strengthen telephone follow-up to assess whether patients are taking their medicines according to the doctor’s advice, which can reduce the occurrence of MD to a certain extent. Follow-up studies should actively explore interventions to reduce MD and improve patient medication safety. This study had some limitations. We recruited patients in a single hospital and reported them by telephone, which may have introduced recall bias. Therefore, multi-center data collection is needed in future studies.

## Acknowledgements

We would like to thank the entire medical staff of the Department of Cardiology of the Sixth People’s Hospital of Nantong, Jiangsu Province.

## Author contributions

**Conceptualization:** Meng-Yao Liang.

**Data curation:** Meng-Yao Liang, Qing-Qing Yang, Li Feng, Wuyang Zhu.

**Formal analysis:** Meng-Yao Liang, Qing-Qing Yang, Li Feng, Wuyang Zhu.

**Investigation:** Meng-Yao Liang.

**Methodology:** Wuyang Zhu.

**Project administration:** Meng-Yao Liang, Qing-Qing Yang.

**Software:** Meng-Yao Liang.

**Writing – original draft:** Meng-Yao Liang, Qing-Qing Yang, Wuyang Zhu.

**Writing – review & editing:** Meng-Yao Liang, Li Feng.
